# Beliefs about Others’ Abilities Alter Learning from Observation

**DOI:** 10.1038/s41598-017-16307-3

**Published:** 2017-11-23

**Authors:** Ida Selbing, Andreas Olsson

**Affiliations:** Division of Psychology, Karolinska Institutet, 171 77 Stockholm, Sweden

## Abstract

Learning what is dangerous by observing others can be safer and more efficient than individual learning. The efficiency of observational learning depends on how observational information is used, something we propose depends on our beliefs’ about others. Here, we investigated how described and actual abilities of another individual (a demonstrator) influenced performance and psychophysiology during learning of an observational avoidance task. Participants were divided into two groups. In each group there were two demonstrators who were described as either high (Described-High group) or low (Described-Low group) in their ability to learn the task. In both groups, one demonstrator had a high ability (Actual-High) and the other had a low ability (Actual-Low) to learn. Participants performed worse in the Described-Low compared to the Described-High group. Pupil dilation, and behavioral data in combination with reinforcement learning modeling, suggested that the described ability influenced performance by affecting the level of attention towards the observational information. Skin conductance responses and pupil dilation provided us with a separate measure of learning in addition to choice behavior.

## Introduction

Learning to avoid harm is key to survival. A core feature in humans and other species is the ability to learn such information by observing the behavior of fellow individuals^1–4^. Although learning which choices to make in order to avoid harm by observing others is often safer than learning by individual trial and error, the usefulness of observational learning depends on both the informational content of the observed behavior as well as if and how this information is used by the observer. Whereas the former is often a function of the behavior of the observed individual, the latter critically depends on the observer’s beliefs and expectations. Here, we examined how prior beliefs about others’ abilities to avoid potentially harmful consequences affect how we learn from those individuals, and how such learning is influenced by their actual performance. To address this fundamental question, we used a novel experimental model of observational instrumental learning in a potentially dangerous environment. We measured behavior and psychophysiological responses, and applied reinforcement learning modelling to examine the mechanisms mediating the impact of prior beliefs and observational information on learning to avoid punishment (mild electric shocks).

Humans often hold prior beliefs about the abilities of others^5^, and these priors might help to direct attention and shape expectations in social situations with relevance for learning. For example, people are more prone to copying the behaviors of others when these are more prestigious^6^. Children copy individuals more if these individuals are proficient, or believed to be proficient, and more if they have a high rather than low status^7^. There is a large body of work arguing for the need for social learning to be selective in order to be adaptive, proposing several possible social learning strategies such as payoff biased and prestige biased learning (see e.g.^8–10^), further supporting the importance of prior beliefs about others’ abilities in social learning. Prior beliefs of someone’s ability can be the result of direct observation of that person’s behavior but impressions are often formed by verbal descriptions^11,12^. However, to what extent the knowledge of someone’s ability improves observational learning from that person depends on if learning is based on copying or on associating the observed choices with their outcomes. As an example, if you are on vacation in a new city trying to figure out at which restaurant to have dinner, copying the observed choices of the experts, i.e. the locals, is probably a better idea than copying other tourists. However, if you base your choice on observation of others’ reactions while eating at a certain restaurant (e.g. expressing content or disgust), then knowing their level of expertise is less useful. Importantly, successful learning here not only relies on having access to valuable observational information about choices and outcomes but also depends on how information is used (e.g. copied or not). From a performance perspective, learning by associating the observed choices with their outcomes, here referred to as observational associative learning, will improve performance regardless of the ability of the observed demonstrator. Learning through copying improves performance only when the demonstrator is more likely than the observer to select the most optimal choice. The need for copying to be selective has led to the proposition of several adaptive social learning strategies such as payoff biased or prestige biased learning^9^, strategies for which there is empirical evidence in both humans^13^ and non-human animals^14,15^. Learning either through observational associative learning or copying comes with specific strengths and weaknesses making the two forms of observational learning more or less suitable in different situations. Observational associative learning would require you to attend to both choices and outcomes; it could be slower and require more effort. Learning by copying can be fast and easy but it can also be vulnerable to environmental changes and relies on the ability of the demonstrator. The pros and cons of the different strategies are thus similar to those often proposed when contrasting copying with asocial, individual, learning^16,17^, where copying is regarded as fast but sensitive to spatial and/or temporal changes and individual learning is regarded as slower but more accurate.

We have previously shown that in an observational learning task where observational information about both the choices and their outcomes is available, participants do not copy, and instead engage in observational associative learning^18^. In this previous study we varied the actual ability of the demonstrator to learn the task and showed that without prior information about the demonstrator’s ability, participants’ performance did not differ as a function of actual ability. Thus, participants appeared to pay equal attention to the available observational information regardless of the demonstrator’s ability when no description of the demonstrator’s ability was given. From a performance optimizing perspective, knowledge about a demonstrator’s ability should not affect observational associative learning since associating a demonstrator’s choice with the outcome is informative regardless of ability. However, learning through copying should be sensitive to such knowledge since the value of copying is directly dependent on the demonstrator’s ability^10,19^. Prior information of an advisor’s level of expertise has been shown to influence advice taking, such that advice given by known experts have greater influence on decisions^20^ and are valued higher^21^ than advice given by novices. In real life, however, prior beliefs about others are not necessarily correct; they can be based on erroneous information, prejudices or simply on non-representative first impressions^22^.

Here, we use a novel experimental paradigm to investigate how the described and the actual abilities of a demonstrator influence observational learning in a simple avoidance task. The assumption that the informational value of a demonstrator’s choices is dependent on the ability of the demonstrator has been argued to lead to beneficial payoff^19^ and prestige^10^ biases in copying of behavior. Previous findings have shown preferential attention to individuals of higher rank during observational learning in chimpanzees^23^. There are also findings pointing to a positivity bias within the ability domain during impression formation^24^. The bias shows that more attention and weight is assigned to information about others that conveyed positive rather than negative ability information (e.g. more weight is assigned to information that someone is skilled rather than unskilled). This is in line with a view of observational social information as more valuable when the person we observe is high, rather than low, in ability. Based on these previous findings we hypothesize that people would pay less attention to the behavior of a demonstrator described as low, as compared to high, in ability. This hypothesis is in line with theories on payoff biased and prestige biased social learning strategies^8^. However, based on our previous findings that participants learn through observational associative learning rather than copying when this is possible^18^, we predict that this decrease in attention would lead to worse performance in the Described-Low compared to the Described-High group. From an objective perspective, information about the ability of the demonstrator should have little or no effect on the level of attention directed towards the demonstrator’s choices and outcomes in the present study, since the ability of the demonstrator has very little effect on the value of observable information in the paradigm we are using. An attentional bias towards skilled or prestigious individuals can however be beneficial in other situations, for instance when learning by copying or in more complex tasks. Evidence of such bias in the present task could indicate that individuals erroneously generalize from other situations.

Varying the actual ability of a demonstrator changes the observer’s learning task; the observational information from a demonstrator behaving randomly faster provides a fuller view of the choice-outcome space while the observational information from a demonstrator that attempts to minimize damage/loss provides a biased view of the choice-outcome space. Furthermore, the choices a demonstrator makes are more predictable when he/she has a high, as compared to a low, ability to learn, making observational learning somewhat more cognitively demanding during observation of a demonstrator with low ability (given that low ability to learn is defined as more random). Based on previous findings^18^ as well as simulations of the task (see SI), we do not expect any effect of actual ability on performance depending on either the difference in informational value or to the difference in predictability. Still, if a demonstrator’s actual ability affects how cognitively demanding the observational learning task is, we might see an interaction between actual ability and level of attention, which we hypothesize is driven by described ability. Thus, when attention is at a sufficiently low level, we could expect an effect on performance driven by how cognitively demanding the task is. However, it is unclear how strong such an interaction between attention, driven by described ability, and cognitive demand, driven by actual ability, would be and under which levels of attention we might see an effect. We are therefore open to the possibility that described ability could interact with actual ability. And consequently, depending on the level of attention, we cannot rule out the possibility of a main effect of actual ability.

Even though copying of poor behavior is counterproductive and should be avoided (but see^25^ for an example showing the surprising efficiency of copying-heavy strategies), a decrease in the ability to observationally learn from the experiences of poor performing others can be dangerous as well. It has been argued that attentionally biased observational learning can explain false beliefs of effective management in organizations^26^ and that similar mechanisms with regards to impression formation could account for the persistence of group stereotypes^27^.

To investigate effects on attention we measured pupil dilation responses which are sensitive to shifts in allocation of attentional resources^28,29^ and the level of cognitive load^30,31^. Pupil dilation responses have been used to measure surprise during learning^32^. We used reinforcement learning modeling to explore in more detail how learning was affected by the described ability. In addition, measures of skin conductance responses, SCRs, which captures changes in autonomic arousal^33^, gaze behavior and pupil dilation responses were used to validate the learning model by serving as psychophysiological indices of learning. Skin conductance responses are commonly used as a measure of learning in humans, often in fear conditioning studies^34^, where the arousal response serves as a proxy for learned fear. Skin conductance responses have also been used to study attentional processes and decision making^35^, for instance to capture the anticipation of an outcome with significant consequence. The inclusion of psychophysiological measures provides us with an additional measure of learning, separate from choice behavior.

## Results

**Figure 1:**
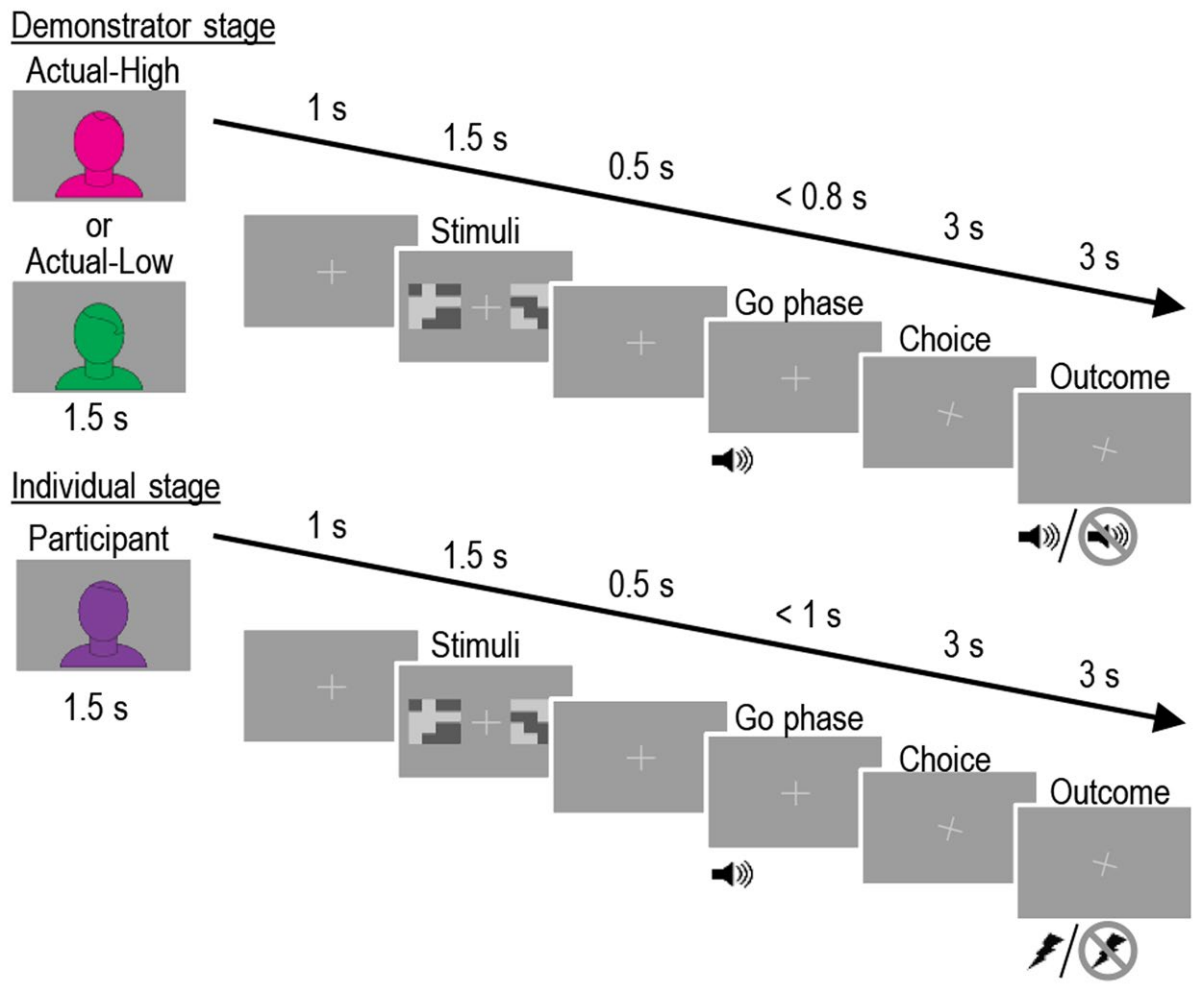
Task Procedure. Each trial was separated into two stages; the Demonstrator stage, during which the participants observed the demonstrator’s choices and outcomes, and the Individual stage, during which the participants made their own choices followed by outcomes. Outcomes were presented either as a shock (Individual stage) or sound indicating shock (Demonstrator stage) or the omission of either shock or sound. The whole session was divided into 5 blocks of 8 trials per demonstrator, giving a total of 80 trials per participant.

Forty-three participants were administered a sequential probabilistic two-choice learning task, in which suboptimal responses lead to increased likelihood of punishment (electric shocks). On each trial (see Fig. 1), participants first observed one of two demonstrators making a choice between two abstract stimuli in order to avoid receiving a shock. Next, participants had to make the same choice themselves. One stimulus (the optimal choice) was associated with a 0.2 probability of being followed by a shock and the other (the suboptimal choice) with a 0.8 probability. To study the role of prior beliefs about the demonstrators’ skills, participants were divided into two groups that received different descriptions about the two demonstrators’ abilities to perform the task. In the ‘Described-High’ group, demonstrators were described as performing the task well and in the ‘Described-Low’ group, demonstrators were described as performing the task poorly. However, in both groups, one demonstrator had a high ability (Actual-High) to learn and performed well while the other performed at random and thus had a low ability (Actual-Low) to learn. This novel 2 (described ability varied between participants: high vs. low) ×2 (actual ability varied within participants: high vs. low) design enabled us to study the unique effects on learning of both the demonstrators’ described and actual abilities, as well as the possible interaction between the same.

### Described ability affected performance

**Figure 2:**
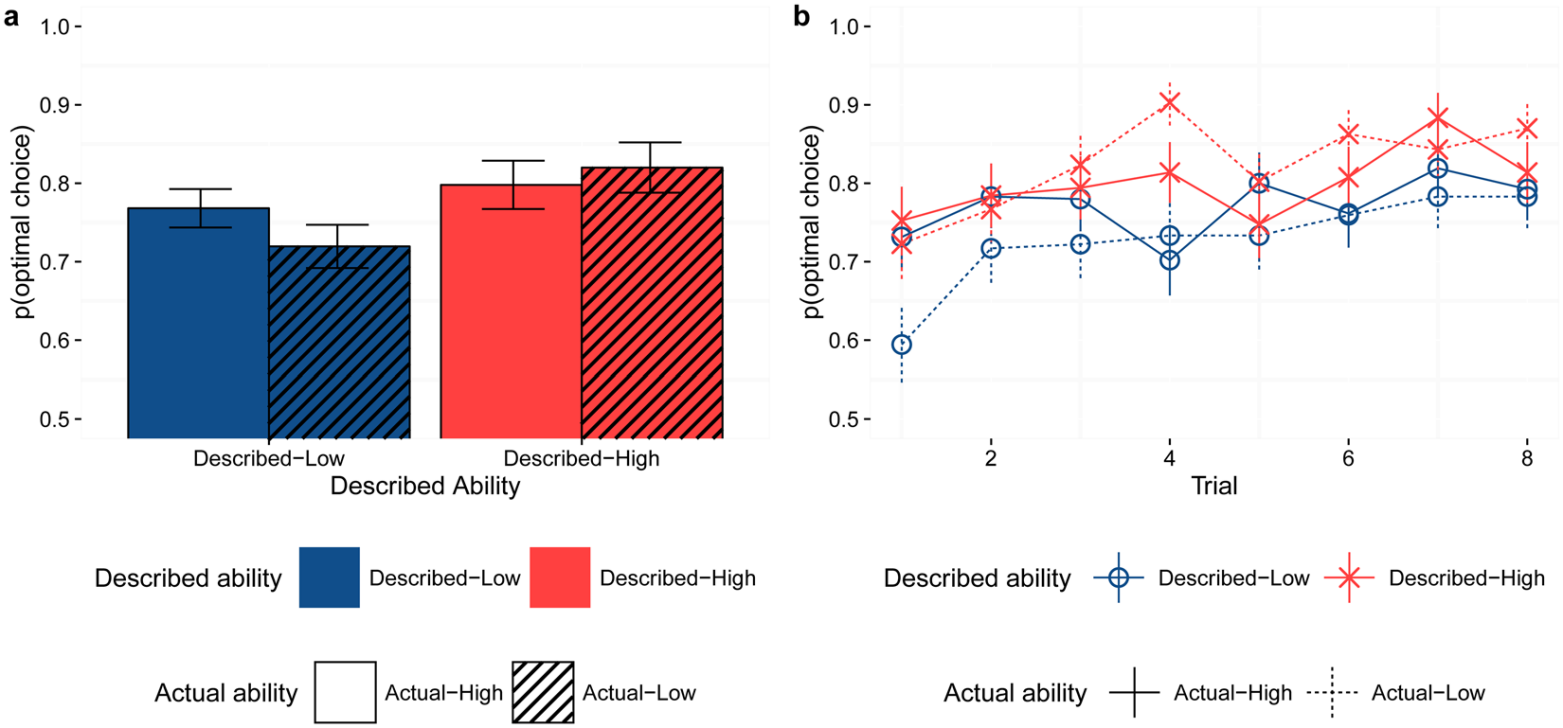
Task Performance. (**a**) Mean performance (defined as the proportion of optimal choices) for each condition. (**b**) Mean performance trial-by-trial for each condition. Performance was lower for the Described-Low group compared to the Described-High group. During observation of the Actual-Low demonstrator performance was lower within the Described-Low group compared to the Described-High group. Error bars represent standard error of the mean.

Using logistic generalized mixed modeling with performance (Optimal/Suboptimal) as dependent variable and maximal random effect structure^36^, as predicted we saw an effect of described ability (*χ* ^2^(1) = 4.82, *p* = 0.028) caused by higher performance for the Described-High group compared to the Described-Low group (*Estimate* = *0.044 SE* = *0.20, p* = *0.028*) as well as a positive effect of trial (*χ* ^2^(1) = 17.44, *p* < 0.001; *Estimate* = *0.14, SE* = *0.03, p* < *0.001*) reflecting the learning curve while there was no effect of actual ability (*χ* ^2^(1) = 0.31, *p* = 0.57; *Actual-Low: Estimate* = *−0.067, SE* = *0.12, p* = *0.57*), see Fig. 2. Including an interaction between described and actual ability in the model did not significantly improve model fit (*p* = *0.083*). By comparing our model against a simple model with trial as the only predictor we showed that our model was significantly better (*p* = *0.011*). An explorative analysis to investigate the effect of sex showed that men performed better than women (*Estimate* = *0.50 SE* = *0.20*, *p* = *0.012*). Including sex as a predictor increased model fit (*p* = 0*.014*) but did not alter our results qualitatively. For detailed model descriptions, analyses and model comparisons see SI. l.

### Described ability influenced the perception of the demonstrators

After each block, participants estimated the number of shocks given to the demonstrator and to themselves. A linear mixed model (LMM) modeling the effects of described and actual ability on the absolute deviation between estimated and actually delivered number of shocks, showed that participants were significantly more accurate during observation of Described-High demonstrators compared to the Described-Low demonstrators when rating the number of shocks delivered to the demonstrator (*χ* ^*2*^(1) = 4.32, *p* = 0.04). This suggests that described ability influenced the level of attention paid to the demonstrators, in turn affecting how accurately they reported the number of shocks given to the demonstrator. To investigate if this could be linked to performance we conducted an LMM, modeling the effect of the absolute deviation between rated and actually delivered number of shocks on mean performance per block. The analysis showed that participants’ performance was worse during blocks where shock estimations were less accurate (*χ* ^*2*^(1) = 5.77, *p* = 0.02). These results further supported our conjecture that described ability affects performance by influencing attention since low attention can be expected to lead to more mistakes in reporting occurred events.

To confirm that the described ability influenced the perceived ability of the demonstrators, an ordered logistic regression analysis showed that Described-High demonstrators were rated higher on performance compared to Described-Low (*χ* ^*2*^(1) = 124.98, *p* < 0.001), in addition to Actual-High demonstrators being rated higher on performance than Actual-Low (*χ* ^*2*^(1) = 8.20, *p* = 0.004). The ratings were carried out after the experiment when participants were asked to rate demonstrator’s performance on a five-graded scale (ranging from 1 = Very poor performance to 5 = Very good performance).

### Pupil dilation responses were sensitive to described ability

We analyzed pupil dilation responses using growth curve analysis^37^ to investigate the effects on attention in our paradigm beyond behavioral choice data (see SI for details). Changes in pupil dilation are, at least partly, regulated by the locus coeruleus believed to mediate allocation of attentional resources^31^ and have been linked to learning processes^32,38^. Changes in pupil dilation responses have been shown to affect the influence of information on existing beliefs^39^. Cognitive control and allocation of attentional resources can be both proactive, in preparation of an upcoming event, and reactive, following an event^40^. We hypothesize that proactive increase of attention as measured by proactive pupil dilation responses would facilitate succeeding processing of information, in our case learning from observation of the demonstrator’s choice and the outcome following the choice.

### Proactive pupil dilation responses were linked to performance

**Figure 3:**
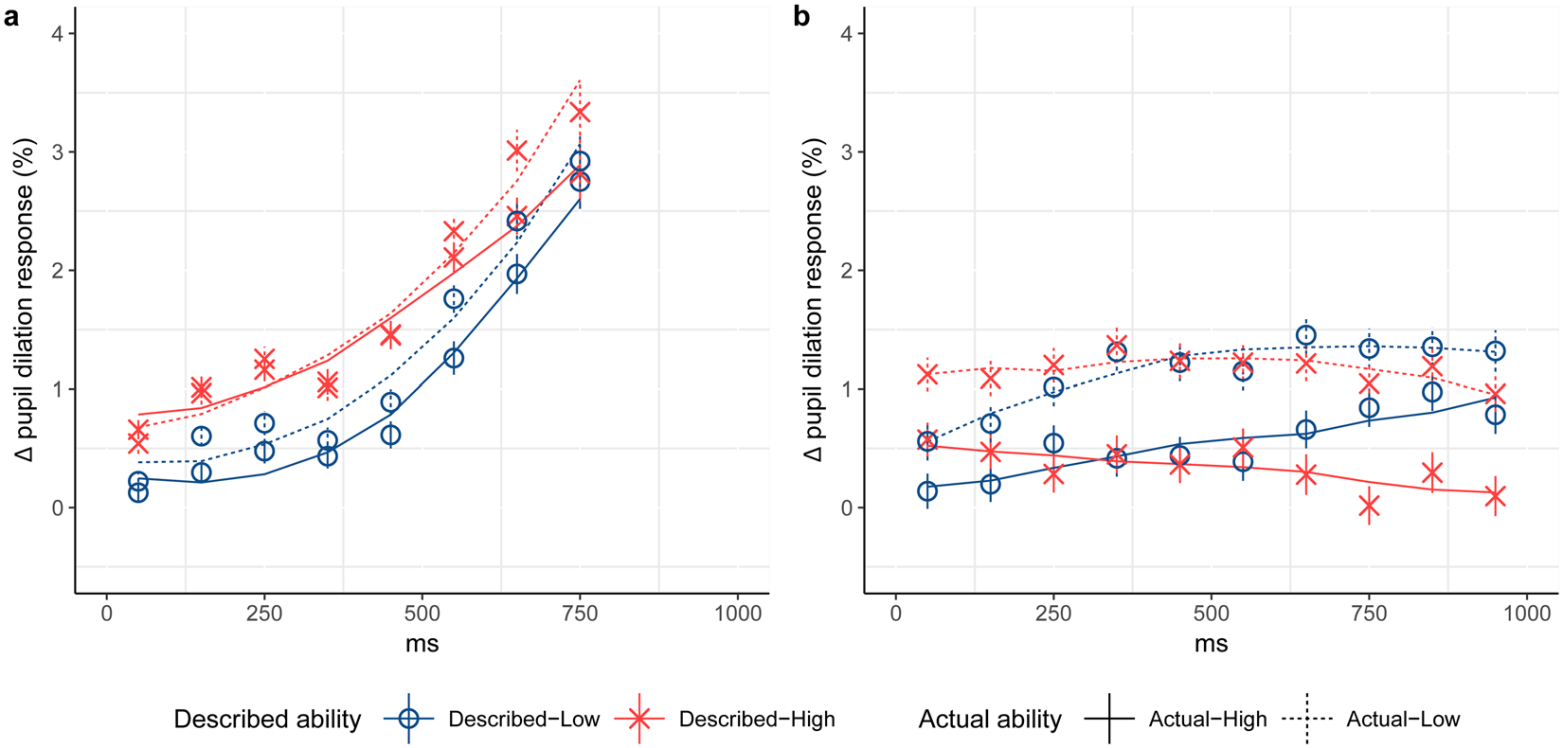
Proactive Pupil Dilation Responses. Data points show empirical data and lines data derived using growth curve analysis. (**a**) The proactive responses in the “go phase” preceding the demonstrators’ choices are higher in the Described-High compared to the Described-Low group. (**b**) The proactive responses in the 1000 ms time-window preceding the demonstrator’s outcome are higher when the demonstrator is Actual-Low compared to Actual-High.

Effects of proactive attention were investigated by analyzing the pupil dilation responses using growth curve analysis, GCA^37^, during the “go phase” preceding the demonstrator’s choice and in the 1s time-window preceding the ensuing outcome. In the”go phase” time-window preceding the demonstrator’s choice, overall pupil dilation responses were larger during observation of the Described-High demonstrator compared to the Described-Low (p = 0.04), see Fig. 3a, indicating a preferential attendance to the choices made by a Described-High demonstrator and supporting the hypothesis that describing the demonstrator’s ability as high increases the level of attention towards that demonstrator. In the time-window preceding the outcome of the demonstrators’ choices overall pupil dilation responses were larger during observation of an Actual-Low compared to an Actual-High demonstrator (p = 0.04), see Fig. 3b, indicating that more attention was directed towards the outcome following a choice made by a demonstrator described as low rather than high in ability. (See SI for model fits and parameter estimates.) Next, we investigated the effect of proactive responses on performance. To do this, we calculated a trial-wise index of attention (Att_total_), a measure of the total amount of proactive attention directed at observational information (i.e. choice and outcome) per trial. Att_total_ was calculated as the sum of the normalized mean pupil dilation responses preceding both the demonstrator’s choice (Att_choice_) and the outcome of that choice (Att_outcome_) (see SI for details). An LMM modeling the effects of proactive attention on performance while controlling for trial order revealed an interaction between Att_total_ and actual ability (*χ* ^*2*^ *(1)* = *6.41*, *p* = *0.01*). Follow-up analyses showed that this interaction was driven by a positive effect of attention, as measured by Att_total,_ on performance during observation of an Actual-Low demonstrator (*β* = *0.088, SE* = *0.059, p* = *0.14*), in contrast to a negative effect during observation of an Actual-High demonstrator (*β* = −*0*.*069*, *SE* = *0.059*, *p* = *0.24*), as would be expected if learning from an Actual-Low demonstrator is more cognitively demanding than learning from an Actual-High. In addition, higher mean Att_outcome_ per block was associated with better accuracy in rating the number of shocks given to the demonstrator in that block (*χ* ^2^ *(1)* = *5.90, p* = *0.02*), further corroborating the interpretation of pupil dilation responses as affected by attention.

To summarize, we first showed that describing the demonstrator as high in ability, rather than low, increased proactive attention preceding the demonstrator’s choice. These results are in line with our hypotheses based on findings that people with a high ability attract more attention than those with a low ability^41^. Secondly, we showed that higher levels of proactive attention preceding both the demonstrator’s choice and the outcome increased performance when the actual ability of the demonstrator was low but not when it was high.

### Reinforcement learning models how described ability affects learning

To investigate the observational learning process on a trial-by-trial basis we analyzed participants’ choices using reinforcement learning (RL) modeling. We used models based on the Q-learning algorithm where the expected value of a choice is updated proportional to the difference between outcome and expected value of the choice, the prediction error, and a learning rate^42,43^. RL modeling allowed us to investigate if and how participants used observational information: if they observed the demonstrator’s choices and outcomes to update the expected values of choices (here observational learning) or if they appeared to copy the behavior of the demonstrators. We fitted twenty-four RL models that differed systematically in how observational information was used, whether or not the model included observational associative learning and/or imitation and whether or not parameters were fitted separately for each within-participant condition (Actual-Low/Actual-High). Models were compared using AIC weights (Akaike Information Criterion weights), which are interpreted as measures of the probabilities of each model being the best, compared to the other models, based on sample predictions on new data^44^ (see SI for details on models and model fits). Model comparisons show strong support for models which included observational learning compared to models which only use individual learning or individual learning paired with copying (sum of mean AIC weights for models which included vicarious reinforcement = 0.923, sum of mean AIC weights for models which did not include vicarious reinforcement = 0.077). From the models which included observational learning, no model clearly stood out as the best model. Based on each model’s mean AIC weight per participant and mean ranking across all participants we choose a simple model of observational learning as the best model. This model included observational learning but no copying of the demonstrators’ choices. According to the model, each choice is represented by a value Q, reflecting the expected outcome of making that choice. During observation of the demonstrator Q-values are updated according to the following equation where *t* denotes the trial number (to clarify that Q-values are updated twice during each trial, the trial number increases with 0.5 during observational learning), α is the learning rate and *outcome* is −1 for shock and 1 for omission of shock:

1 $${Q}_{choice}(t-0.5)={Q}_{choice}(t-1)+\alpha \ast (outcom{e}_{demonstrator}-{Q}_{choice}(t-1))$$

Next, the softmax activation function uses the Q-values to calculate the probability of making each choice. The choice associated with a higher Q-value will have a higher probability of being chosen but this is controlled by the inverse temperature parameter β, regulating how deterministic choices are (where low values indicate highly deterministic choices). The outcome following individual choices are used to update the Q-values again:

2 $${Q}_{choice}(t)={Q}_{choice}(t-0.5)+\alpha \ast (outcom{e}_{individual}-{Q}_{choice}(t-0.5))$$

Note that Q-values are similarly updated, using the same learning rate, regardless of whether or not the outcome follows the demonstrator’s or the individual’s choice. In this model we have two free parameters, *α* and *β*, which we fitted to participants choices. The learning rate parameter *α* reflects how fast expected outcomes for available choices are updated. Suboptimal performance can arise from both exceedingly high and low learning rates, resulting in too much or too little weight assigned to the latest piece of information. The inverse temperature parameter *β* is often interpreted as a measure of the tendency to explore but is more correctly understood as a measure of how noisy choice-behavior is.

Next, to further explore the mechanisms of the learning processes we analyzed the distributions of these fitted parameters. We did this by looking at how described ability affected the distribution of fitted parameters as categorized according to a cluster analysis of parameter combinations (see SI). The Described-High group appeared to have more fitted parameters belonging to a cluster with low values of both α and β while the Described-Low group had medium to high values of α and low to medium levels of β. Although this pattern was not significant (p = 0.16) our findings could indicate that described ability possibly affects both how well observed information is integrated over time as well as how noisy choices are. In our experiment, poor performance could thus be caused both by an impaired ability to integrate information over time in addition to choices simply being more random. However, it is not always straightforward to interpret fitted parameter values in RL models since it is difficult to distinguish the separate impacts on performance of the α and β parameters^45^ and therefore wish to caution against relying too heavily on this finding. To validate the RL model we showed that often used measures of surprise and learning in the form of reactive pupil dilation responses^32,38^ and skin conductance responses which measure arousal responses^34,35^ were sensitive to model derived observational prediction errors, both following the demonstrators’ choices and the outcome of those choices. Pupil dilation and skin conductance responses were larger following more surprising events associated with higher absolute prediction errors. In addition, gaze patterns during presentation of the choices reflected the model derived certainty of which choice was the optimal such that participants looked more at the optimal choice the larger the difference in expected value between the optimal and suboptimal choice, which is in line with studies showing that gaze is directed to the preferred choice^46,47^. (See SI for details.)

## Discussion

In the present study, we used a novel experimental paradigm to examine how prior beliefs about others’ ability impact learning from these individuals. Participants learned to avoid punishment (mild electric shocks) through observation of demonstrators described as having either a high (Described-High) or low (Described-Low) ability to learn. We also investigated if the described ability interacted with the actual ability of the demonstrators by varying how well they learned the task. Each participant observed two demonstrators, one that learned quickly and performed well (Actual-High) and one that behaved randomly and performed poorly (Actual-Low).

Our results show that people’s prior beliefs about others’ ability as low can alter observational learning. Learning from observing demonstrators, who were described as low in ability, resulted in worse performance as compared to observation of demonstrators described as having a high ability. Notably, we have previously shown that actual ability does not affect performance in a similar paradigm where no description of ability was given^18^; we therefore did not expect any main effect of actual ability. Our current results extend these previous results by showing the impact of prior beliefs on observational learning. These results are especially interesting since the actual ability of the demonstrator does not significantly affect how useful or valuable it is to learn by observation the choices and outcomes of that demonstrator in the present paradigm. Information about the level of ability should theoretically therefore not matter to the participant. We further demonstrated that describing demonstrators’ abilities as low compared to high also led to less accurate estimations of the number of shocks that the demonstrators had received. Moreover, results from analyses of pupil dilation responses, previously associated with attention^31,48^, indicated that the level of proactive attention before observation of a demonstrator’s choice was lower during observation of a demonstrator described as having low, compared to high, ability.

We further observed a trend towards an interaction between described and actual ability driven by a greater difference in performance during observation of demonstrators that performed poorly. We hypothesize that this could be driven by a difference in how cognitively demanding the observational learning task is, which depends on actual ability, interacting with the level of attention during observation, which depends on described ability. The choices of a demonstrator with actual low ability, who is making random choices, are more difficult to predict and learning from a demonstrator with low ability would therefore require more attention than learning from a demonstrator with high ability. This could make observational learning from a demonstrator with low ability more susceptive to changes in attention. In support of this argument we showed that the effect of proactive attention on performance differed as a function of the actual ability of the observed demonstrator. Higher proactive attention was linked to better participant performance when the demonstrator had an actual low ability to learn (random choices). When the demonstrator had an actual high ability to learn we saw the opposite pattern where instead lower proactive attention was linked to better participant performance. Further research is needed to investigate this potential interaction between described and actual ability on observational learning.

We used RL modeling to investigate and describe the effects on learning in more detail. RL modeling showed clearly that participants in both groups used observational information to learn and that neither of the groups appeared to copy the behavior of either of the demonstrators to any large extent. This finding can be related to previous findings showing that payoff biased learning is adaptive but under-used^49^. The model which best explained our behavioral data was a simple model which included two free parameters, a learning rate which was the same for learning from own outcome as well as from the demonstrators’ outcome and a parameter which regulates how deterministic choices are depending on the learned expected values of each choice. A closer look at the distributions of the fitted parameter values in the best model suggests that described ability affected both the learning rate and how deterministic choices were. The learning rate was higher and choices were less deterministic in the group that observed demonstrators described as low in ability. A high learning rate could indicate a failure to integrate information over time, possibly as a result of low cognitive effort or poor working memory^45^. Relatively non-deterministic choices it also what we would expect if the participants failed to notice which choice a demonstrator just made. It is however difficult to separate the parameters’ specific contributions to behavior^50,51^. To validate our model we analyzed effects of observational prediction errors on psychophysiological data. Skin conductance and reactive pupil dilation responses were sensitive to observational prediction errors and gaze patterns were sensitive to the difference in expected value between both choices. Taken together, our behavioral results, pupil dilation data and RL modeling, support our hypothesis that a demonstrator’s described ability affects the level of attention that the participants direct towards the available observational information.

The present study does not answer the question of why described ability would affect the level of attention or cognitive effort of an observer. We propose that the participants in the group where the demonstrators are described as high in ability allocated more attention because high ability should be more informative in a very general sense, and more diagnostic of an individual’s character than low ability^52^. It is also possible that participants concluded that since the choices of demonstrators that were low in ability themselves were not informative or useful to copy, paying attention to the choices of those demonstrators would not be useful. Participants would then behave according to a heuristic based on prestige or payoff biased learning^8,10^. This argument is supported by our analysis of the proactive pupil dilation responses which indicate that the described ability affects attention directed at the choices made by the demonstrators, not necessarily the outcome of those choices. However, participants in the Described-Low group also made more mistakes when attempting to estimate the number of shocks given to the demonstrator during a block which could be explained by a difference in the level attention directed toward the outcome as well. It is important to note that these arguments which are based on beliefs of the value of observational information rely on participants misconceiving the learning task. The value of observational information is in fact slightly higher when a demonstrator makes random choices, constantly exploring the environment; rather than making choices that are biased towards the optimal choice (see SI).

In line with theories on attention as a utility maximizing system, which mediates search for information^53^, proactive or preparatory, attention has been shown to be sensitive to the prospect of reward^54,55^, such that more attention is given to information with greater value. These previous studies support our interpretation of the current results that prior beliefs of demonstrators’ ability affected how participants evaluated observational information and, as a consequence of that, how much attention they directed to the demonstrators. It is interesting that participants would have evaluated the observational information differently depending on the description of the demonstrator when in fact learning benefits greatly from using observational information regardless of the demonstrator’s actual ability (and where, if anything, observational information from a poor performing demonstrator is actually slightly more valuable). One explanation could be that this bias, to attend to the behavior of supposedly skilled or successful others at the cost of failing to learn from presumably poor performing individuals, would be adaptable in certain (possibly more ecologically valid) environments. Consider for instance the task of learning how to build a chair. The task involves several steps as well as several solutions and the quality of the chair depends on how each step is performed. Observational learning would in this case only be efficient if the observed demonstrator is at least close to building a proper chair. Learning from someone randomly trying out ways to assemble different pieces of wood would be extremely slow. It has been suggested that such narrow-peaked search landscapes, where only behavior close to a local optima, generates valuable feedback about the location of the solution, could increase tendencies to copy^56^.

Our results show that something as simple as a short description of someone else can lead to impairments in learning simply by a decrease in attention to valuable observational information. Systematically attending to the behavior of those that are described as learning and performing well is a form of biased selective sampling. This mechanism can be tied to varying occurrences of illusory correlations, such as stereotypes^57^ and biased organizational theories^26^ which show that undersampling of failure leads to false beliefs regarding the nature of effective management. We have shown here that biased sampling of others’ behavior can give rise to suboptimal learning and that the problem can worsen as a function of the nature of the observed behavior.

## Materials and Methods

### Participants

A total of 46 healthy participants were recruited and paid for participation in the experiment approved by the Regional Ethical Review Board in Stockholm and the experiment was performed in accordance with relevant guidelines and regulations. Three participants were excluded due to technical issues. The remaining 43 participants were randomly assigned to either the Described-Low or Described-High group (Described-Low: n = 21, 14 women, mean age 24.5 y [sd = 6.9]; Described-High: n = 22, 14 women, mean age 24.9 y [sd = 4.9]). Before the experiment, all participants signed an informed consent form.

### Experimental Procedure

Participants performed a probabilistic two-choice task to learn to avoid shocks which included observation of a demonstrator, adopted from Selbing *et al*.^18^. We choose an aversive learning task since it is theorized that social learning should be more advantageous when negative consequences can be costly (59). Participants were told that the demonstrators they were to observe had been participants in a previous, similar, experiment and that these previous participants had been categorized as either high or low in their ability to learn the task. Participants were randomly assigned to one of two groups; participants in the Described-Low group were told that the demonstrators had been categorized as low in ability, and those in the Described-High group were told that the demonstrators had been categorized as high in ability. However, in order to vary actual ability within participants, the two demonstrators that each participant observed varied in their ability to learn the task (unbeknownst to the participants): the Actual-High demonstrator learned quickly and performed the task well, the Actual-Low demonstrator performed randomly throughout the experiment. The choices of the demonstrators were controlled by a preprogrammed reinforcement learning algorithm and the mean probabilities of optimal choice were 0.81 and 0.50, respectively (see SI for details). The variability in the demonstrators’ behavior (i.e. the Actual-High demonstrator did not always make the optimal choice and the Actual-Low demonstrator did not always make the suboptimal choice) made the task more similar to real-life behavior and corresponds to the behavior we expect from a real participant (that either has a high or low ability to learn).

Apart from an initial training block, participants completed five blocks of eight trials per demonstrator, resulting in a total of 80 trials (2 × 5 × 8). For each block the participants had to repeatedly choose between two randomly drawn pictures of equal luminance, one assigned to be the optimal choice (probability of being paired with a shock = 0.2) while the other was the suboptimal (probability of being paired with a shock = 0.8). Each trial in the setup consisted of an initial demonstrator stage during which the demonstrator made a choice followed by outcome before the individual stage during which the participant made a choice followed by an outcome. The sequence of events was the same for both stages, see Fig. 1. Each stage began with a 1.5 s presentation of a figure indicating whose turn it was. Next, a fixation cross was displayed and after 1 s the choice stimuli was presented for 1.5 s. Half a second after the presentation of the choice stimuli a “go-sound” was played signaling that it was time to make a choice. During the individual stage the sound lasted a maximum of 1 s or until a choice was made. During the demonstrator stage the duration of the sound was randomized between 300–800 ms to simulate termination following the demonstrator’s choice. The fixation cross was then rotated 20° for 6 s to indicate which choice was made (right: clockwise: left: counterclockwise). If the consequence of the choice was a shock (100 ms DC-pulse, individually set to be unpleasant but not painful) this was administered after 3 s either directly to the participant (individual stage) or indicated by a short neutral “shock-sound” (demonstrator stage). At the end of each block, participants were asked to estimate the number of administered shocks. After finishing the experiment, participants filled out a set of questionnaires (see SI).

### Data acquisition

The experiment was presented and behavioral data collected using E-Prime (Psychology Software Tools). In addition to measure learning as choice behavior we also recorded psychophysiological measures of learning: gaze, pupil dilation and skin conductance responses. Pupil dilation data is commonly used in learning paradigms to measure surprise and attention^31,58^ and skin conductance responses are often used as a measure of learning in conditioning paradigms^34^ and has been linked to attentional processes in decision making tasks as well^35^.

Eye tracking data with a resolution of 50 Hz was collected through iViewX 1.6 using an SMI remote Red III eye tracker placed on the desk in front of the participants. Eye tracking data from 7 participants was excluded due to poor data quality leaving 36 participants to be included in analyses of gaze patterns and pupil dilation responses. Skin conductance data was collected using a pair of Ag-AgCl electrodes attached to the index and middle finger of the left hand. The signals were amplified and recorded at 250 Hz using BIOPAC Systems (Santa Barbara, CA). Skin conductance data from 2 participants were excluded to poor quality leaving 41 participants to be included in further analyses. For additional details on material, data acquisition and preparation see SI.

### Data availability

The datasets generated and analyzed in the current study are available from the corresponding author on reasonable request.

## Electronic supplementary material

Supporting Information
