# Supplementary material for: Beliefs about Others’ Abilities Alter Learning from Observation
Source: Sci Rep. 2017 Nov 23;7:16173. doi: 10.1038/s41598-017-16307-3 (PMC5701038; doi:10.1038/s41598-017-16307-3)
Supplement: Supplementary file 1 — Supporting Information [file 41598_2017_16307_MOESM1_ESM.pdf]

Supporting Information

Beliefs about Others' Abilities Impair Learning from Observation

Ida Selbing[a,*], Andreas Olsson[a]

[a] Division of Psychology, Karolinska Institutet, 171 77 Stockholm, Sweden

[*] To whom correspondences should be addressed: ida.selbing@ki.se

1. SI Material & Methods

1.1. Stimuli

Before each phase, simple monochrome figures/avatars were used to index whose turn it was to make a choice, the participant's or one of the two demonstrators. The participants initially selected which avatar out of 9 possible that would represent them during the task, and the demonstrators' avatars were randomly selected from those remaining. The experiment was shown on a 24˝ screen with resolution 1600x1200 pixels. Participants watched the screen from a distance of approximately 60 cm.

The pictures used as choice stimuli consisted of 40 randomly generated geometric figures of equal luminance. For each participant, 22 pictures were randomly drawn and randomized into 11 pairs in which one stimulus was randomly selected to be the optimal while the other was the suboptimal. Of these pairs, one was used in the training block and the rest in the remaining 10 blocks.

To minimize alterations in pupil dilation due to changes in the presentation of visual stimuli on the screen during the experiment, auditory stimuli was used to signal when it was time for the demonstrator or participant to make a choice, and to indicate that the demonstrator received a shock. These auditory stimuli are referred to as the "go-sound" and "shock-sound". The "go-sound" consisted of a 1 second long D6 sinus tone while the "shock-sound" was a 232 ms long non-aversive beep oscillating around C6. The sounds were played to the participants using headphones.

1.2. Demonstrators' behavior

Demonstrators' actual ability was manipulated by allowing a simple RL-algorithm to control the behavior of the Actual-High demonstrators while the Actual-Low demonstrators always choose

randomly. The RL algorithm controlling the behavior of the Actual-High demonstrators was based on the Q-learning algorithm (1) were choices were assigned to Q-values representing the expected outcome of making the choice. At the start of each block Q-values were set to 0. Choices were made based on the softmax activation function which calculates the probability of making either choice, here denoted A and B:

$$p_A(t) = \frac{\exp(Q_A(t)/\beta)}{\exp(Q_A(t)/\beta)+\exp(Q_B(t)/\beta)} \quad [1]$$

$$p_B(t) = 1 - p_A(t) \quad [2]$$

where the inverse temperature β was set to 0.4. The outcome of a choice was used to calculate a prediction error, δ_{choice}, the difference between the expected and predicted outcome (the outcome of a shock was set to -10, and the outcome of a omitted shock was set to 10). The prediction error was used to update the Q-values :

$$Q_{choice}(t) = Q_{choice}(t-1) + \alpha * \delta_{choice} \quad [3]$$

where the learning rate α was set to 0.3. See SI fig 1 for a visualization of the demonstrator's performance across the experiment.

1.3. How the value of observational information depends on demonstrator behavior

To investigate how the ability of the demonstrator might affect the value of observational information, we simulated participants undergoing the experiment and learning through observation of a demonstrator that either learned well or made random choices. The simulated participants learned the task by pairing the observed choices with observed outcomes in addition to making choices themselves and learning from them. Simulating ten thousand blocks showed a small advantage, of learning from a demonstrator with low ability (increased probability of making the optimal choice approximately 0.029), see SI fig 2.

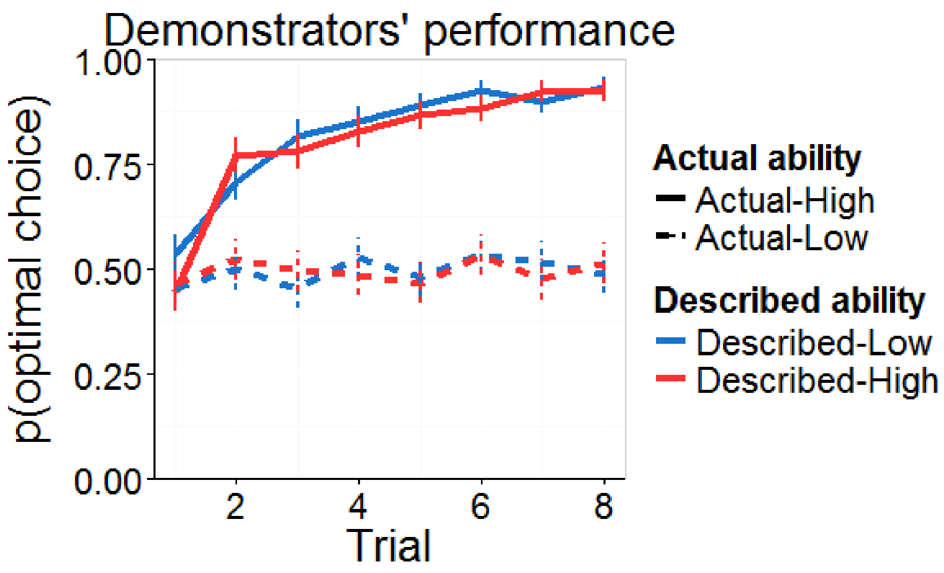

SI Fig. 1. Demonstrators' performance over trials across the experiment. Actual-High demonstrators learned quickly while Actual-Low demonstrators' performed around chance throughout.

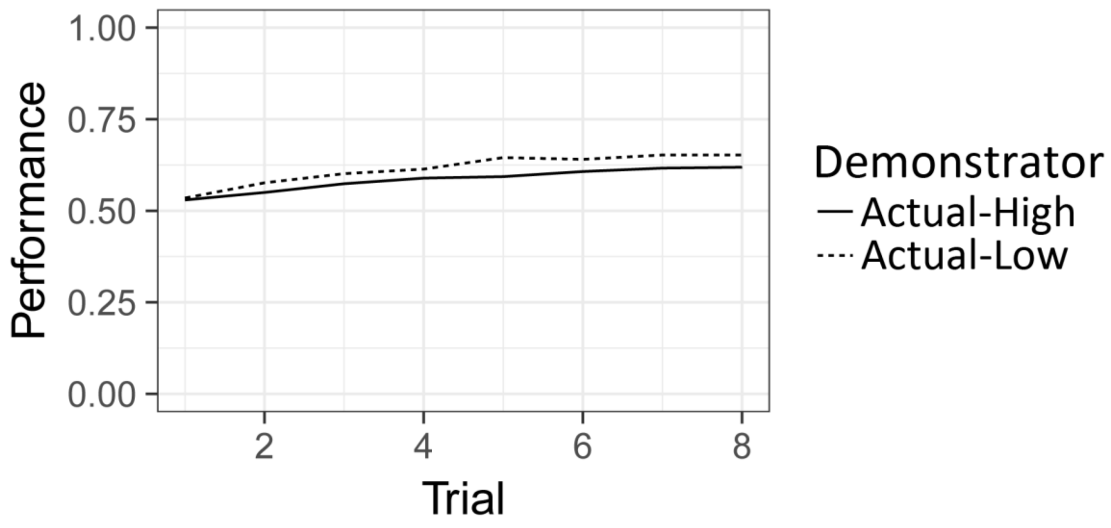

SI Fig. 2. Mean performance per trial of ten thousand simulated blocks of observational learning from either a demonstrator with either a high or low ability to learn combined with individual learning. Results show a small advantage of learning from a demonstrator with actual low ability.

1.4. Instructions

Participants were instructed that they were to observe the behavior of previous participants in a similar experiment. They were further told that the behavior of these previous participants had been observed and evaluated in order to divide them into two groups of equal sizes based on their level of ability, either high (Described-High) or low (Described-Low). The information regarding how the evaluation had been done was very vague, and it was not stated that the evaluation necessarily relied on any objective measure of performance (e.g., number of shocks received).

Participants were instructed that the probabilities of receiving a shock based on choice were the same for both them and the previous participant although making the same choice as the demonstrator in a trial were the demonstrator did not receive a shock not necessarily resulted in avoidance of shock for themselves.

1.5. Questionnaires

Following completion of the experiment, participants were asked to fill out a set of questionnaires to asset personality traits that could possibly influence the efficiency of observational learning. Anxiety was measured both as state and trait using the State-Trait Anxiety Inventory (STAI). Empathic concern, fantasy and perspective taking were measured using the Interpersonal Reactivity Index (IRI). Social conformity was measured using a subset of the questions in the Swedish universities Scales of Personality (SSP).

2. Data acquisition and preparation

2.1. Eye-tracking

Blinks were identified as instances when the diagonal pupil width was 0 for 50-400 ms and pupil widths (diagonal and horizontal) during blinks ±60 ms were linearly interpolated. Pupil data was

filtered using a 10 Hz low pass filter. Pupil diameter was calculated as the average pupil width based on the diagonal and horizontal measures. Pupil dilation responses were calculated as the relative change in diameter compared to the mean pupil size during the 0.5 s presentation of the fixation cross following the stimuli presentation but before the "go-phase".

2.2. Skin Conductance – preparation and scoring

Skin conductance data was analyzed using the AcqKnowledge software (BIOPAC Systems). Raw data was filtered using a 1 Hz low pass filter and a 0.01 Hz high pass filter. Responses were scored as the first peak-to-peak response starting within a time-window of 0.5-4.5 s following the onset of each event of interest (observed choice, observed/heard outcome). Responses were normalized within subject.

3. Analyses

3.1. Behavioral analyses

Performance was analyzed using a Logistic Generalized Mixed Model (LGMM) which modeled the effect of described ability and actual ability as well as trial (centered) on choice, categorized according to optimality (optimal/suboptimal). We used by-participant random intercepts and random slopes of actual ability within participant. The reported main- and interaction effects were evaluated with "Type II" analysis of deviance tests based on the Wald statistic.

The effect of the described ability on the absolute deviation between estimated and actually delivered number of shocks during a block was analyzed using a Linear Mixed Model (LMM) with by-participant random intercept. We further analyzed the effect of the absolute deviation per block on mean performance per block using an LMM with by- participant random intercept and

random slopes of the absolute deviation within subject. The reported main- and interaction effects were evaluated with "Type II" analysis of deviance tests based on the Wald statistic.

3.2. Pupil dilation

We analyzed the pupil dilation responses using growth curve analysis, GCA (2). For the proactive pupil dilation responses we analyzed orthogonal linear and quadratic changes in pupil dilation over time (first- and second-order polynomials of time). For the reactive responses, we also included cubic changes in pupil dilation over time (third-order polynomial) to our analysis. Participant and participant-by-condition random effects were used on all time terms. For the proactive responses we analyzed fixed effects of described ability and actual ability on all time terms while for the reactive responses we analyzed fixed effects of the observational prediction errors on all time terms. The effect of the observational choice prediction error, see [8], was analyzed for all trials and the effect of the observational prediction error, see [4], was analyzed for trials where the demonstrator received a shock (since no-shock trials did not include any time-locked event signaling the omission of shock).

3.3. Gaze patterns

We analyzed gaze direction during stimuli presentation, for both the observational stage and the individual stage, using two areas of interest (AOIs) defined as Optimal/Suboptimal stimuli. The AOIs consisted of two areas (193 pixels x 192 pixels) centered round the two stimuli. Gaze direction was analyzed using logistic GCA with the AOI Optimal as the target. We included orthogonal linear, quadratic and cubic changes over time (first, second and third order polynomials). We first analyzed the fixed effects of the time terms only to investigate the probability of directing gaze towards the optimal choice over time. Secondly, to investigate if this probability would be affected by a trial-by-trial measure of the certainty that the optimal choice was the optimal, we analyzed the fixed effects of a certainty parameter (derived from our winning RL model) on all time terms for the separate stages (observational/individual). The certainty parameter was defined as the difference between the expected values of the optimal and the suboptimal choices, $certainty = Q_{optimal} -$

$Q_{suboptimal}$. Thus, expecting a high value from the optimal choice and a low value from the suboptimal choice would lead to high certainty (see SI Model description for additional information regarding the expected values). All models used by-participant random intercepts and random slopes of all main effects within participant (a full random structure was not possible in the models which included certainty as a predictor due to convergence problems).

3.4. Skin conductance responses

We analyzed the effect of the observational choice prediction error, see equation [8], and the observational prediction error, see equation [4], on skin conductance responses, SCRs, using LMMs. The models included fixed effects of the normalized observational prediction error of interest and of trial number. The models included by-participant random intercepts and random slopes for all variables within participant, i.e. a full random structure (3).

4. Reinforcement Learning

4.1. Model description

We formulated several reinforcement learning models that differed in their use of observational learning parameters. Here we describe the general structure of the models. All models are based on the Q-learning algorithm were choices (A,B) are associated with Q-values, expected values, initially set to 0. These values are updated following a choice paired with an outcome. Each trial starts with observation of a demonstrator making a choice followed by an outcome. The model updates the Q-values based on the observational prediction error $\delta_{obs\ outcome}$:

$$\delta_{obs\ outcome}(t-1) = Q_{obs.\ choice}(t-1) - outcome_{observed} \quad [4]$$

$$Q_{obs.\ choice}(t-0.5) = Q_{obs.\ choice}(t-1) + \alpha_{obs.} * (\delta_{choice}(t-1)) \quad [5]$$

The softmax activation function is used to calculate the probability of making each choice (A,B) based on the Q-values and the inverse temperature parameter β.

$$p_A(t-0.5) = \frac{\exp(Q_A(t-0.5)/\beta)}{\exp(Q_A(t-0.5)/\beta)+\exp(Q_B(t-0.5)/\beta)} \quad [6]$$

$$p_B(t-0.5) = 1 - p_A(t-0.5) \quad [7]$$

The probabilities of making each choice can then be influenced by imitation in proportion to an imitation parameter κ and the observational choice prediction error $\delta_{obs\ choice}$:

$$\delta_{obs\ choice}\,(t-0.5) = 1 - p_{obs.\ choice}(t-0.5) \quad [8]$$

$$p_{obs.\ choice}(t) = p_{obs.\ choice}(t-0.5) + \kappa * (\delta_{obs\ choice}(t-0.5)) \quad [9]$$

$$p_{\neg obs.\ choice}(t) = 1 - p_{obs.\ choice}(t) \quad [10]$$

After an individual choice is made the Q-values are updated again based on the individual prediction error $\delta_{ind\ outcome}$:

$$\delta_{ind\ outcome}(t-0.5) = Q_{ind.\ choice}(t-0.5) - outcome_{individual}) \quad [11]$$

$$Q_{ind.\ choice}(t) = Q_{ind.\ choice}(t-0.5) + \alpha_{ind.} * (\delta_{ind\ outcome}(t-0.5)) \quad [12]$$

All models included the individual learning rate $\alpha_{ind.}$ as a free parameter. Based on this setup we constructed a set of models which varied in how the observational learning rate $\alpha_{obs.}$, imitation parameter κ and inverse temperature parameter β were defined:

- $\alpha_{obs.}$ was either i) fixed to 0, ii) set to $\alpha_{ind.}$ iii) one free parameter α_s or iv) two free parameters $\alpha_{s-AL}, \alpha_{s-AH}$ to allow for differences in learning rates depending on actual ability of the demonstrator
- κ was either i) fixed to 0, ii) one free parameter κ or iii) two free parameters κ_{AL}, κ_{AH} to allow for differences in copying depending on actual ability of the demonstrator

- β was either i) one free parameter or ii) two free parameters β_{AL}, β_{AH} to allow differences in choice selection depending on the actual ability of the demonstrator.

This allowed us to test 4 x 3 x 2 = 24 different models, see SI Table 1.

4.2. Model fitting & model comparison

The free parameters for each model were individually fitted for each participant over all trials using by minimizing the negative log-likelihood, $-ln(L)$, of each model. This was done in R (4) using the mle2 function from the bbmle package employing the optim optimization function and the BFGS optimization method. Each set of parameters was fitted 10 times with randomized initial parameters after which the best fitted parameters were chosen, in order to avoid local minima. The learning rate parameters and imitation rate parameters were constrained within the interval [0,1] while the inverse temperature parameters were constrained within the interval [0,5].

Model comparisons were carried using the AIC weights, $wAIC_i$, for each model and participant (5). AIC weights are calculated from the AIC values that measure the goodness of fit of a model while also taking into account its complexity:

$$AIC = 2k - 2\ln(L) \qquad [13]$$

where k is the number of fitted parameters and $-ln(L)$ is the negative log-likelihood. For each model i ΔAIC_i is calculated as $\Delta AIC_i = AIC_i - AIC_{min}$ were the AIC_{min} is the AIC value for the best model (i.e. the model with the lowest AIC value) for that participant. AIC weights are then calculated by comparing the AIC values for all models of interest over each participant:

$$wAIC_i = exp\left(\frac{-\Delta AIC_i}{2}\right) / \sum_{m=1}^{M} exp\left(\frac{-\Delta AIC_m}{2}\right) \qquad [14]$$

where *M* denotes the number of models compared. AIC weights provide a measure of the weight of evidence for each model in a given set of candidate models. We compared models by looking at the mean $wAIC_i$, mean rank order and number of wins per model across participants and separated by groups (Described-High/Described-Low), see SI table 1.

5. SI Results

5.1. Behavioral results

The significant interaction between described ability and actual ability was followed by pairwise comparisons adjusted for multiple comparisons using Bonferroni corrections. This revealed a significant difference between the Described-High/Actual-Low and Described-Low/Actual-Low conditions only ($\beta=0.71$, $SE=0.26$, $Z=2.71, p=0.041$).

5.2. Proactive Pupil dilation responses

Results from the F tests (Type II Wald Chisquare tests) of the GCA on proactive pupil dilation responses in the "go phase" preceding the demonstrator's choice showed significant effects of the linear time term, ot1 ($\chi^2(1) =29.44$, $p<0.01$), quadratic time term , ot2 ($\chi^2(1) =7.60$, $p<0.01$)and the described ability ($\chi^2(1) =4.04$, $p=0.04$). See SI table 2 for parameter estimates. Results from the F tests (Type II Wald Chisquare tests) of the GCA on proactive pupil dilation responses in the 1 s time-window preceding onset of the presentation of the outcome of the demonstrator's choice showed a significant effect of the actual ability ($\chi^2(1) =4.22$, $p=0.04$). See SI table 3 for parameter estimates.

	Estimates	Std.Error	t
Intercept	1.706	0.306	5.582
ot1	2.351	0.619	3.796
ot2	0.576	0.349	1.651
Described ability	-0.094	0.426	-0.168
Actual ability	0.023	0.279	0.084
ot1:Described ability	-0.524	0.862	-0.607
ot2:Described ability	0.150	0.484	0.309
ot1:Actual ability	0.086	0.485	0.177
ot2:Actual ability	-0.044	0.321	-0.136
Described ability:Actual ability	0.350	0.389	0.900
ot1:Described ability:Actual ability	0.109	0.675	0.162
ot2:Described ability:Actual ability	-0.151	0.445	-0.340

SI Table. 2. Parameter estimates of the proactive pupil dilation response in the “go phase” preceding the presentation of the demonstrator’s choice. Described-High is coded as 0, Described-Low is coded as 1. Actual-High is coded as 0, Actual-Low is coded as 1.

	Estimates	Std.Error	t
Intercept	0.254	0.589	0.431
ot1	-0.383	0.443	-0.864
ot2	-0.016	0.195	-0.084
Described ability	0.123	0.821	0.150
Actual ability	0.0887	0.438	2.026
ot1:Described ability	1.009	0.617	1.635
ot2:Described ability	0.043	0.268	0.161
ot1:Actual ability	0.246	0.425	0.578
ot2:Actual ability	-0.223	0.266	-0.840
Described ability:Actual ability	-0.137	0.611	-0.224
ot1:Described ability:Actual ability	-0.045	0.591	-0.075
ot2:Described ability:Actual ability	-0.173	0.366	-0.471

SI Table. 3. Parameter estimates of the proactive pupil dilation response in the 1 s time-window preceding the presentation of the outcome of the demonstrator’s choice. Described-High is coded as 0, Described-Low is coded as 1. Actual-High is coded as 0, Actual-Low is coded as 1.

5.3. Reactive Pupil dilation responses

The reactive pupil dilation responses when participants observed the demonstrator's choices were greater when the model derived observational choice prediction error was large, i.e. more surprising, se SI Fig. 3 A. The effects of the observational choice prediction error on all three time terms, the linear, quadratic as well as the cubic were significant (ot1:Observational Choice PE: $\chi^2(1)$ =9.99, p<0.01; ot2: Observational Choice PE: $\chi^2(1)$ =12.70, p<0.01; ot3: Observational Choice PE: $\chi^2(1)$ =9.58, p<0.01). In addition, there was an effect of the cubic time term, ot3, only ($\chi^2(1)$ =40. 08, p<0.01). See SI table 4 for parameter estimates.

	Estimates	Std.Error	t
Intercept	2.813	0.663	4.245
ot1	0.612	4.505	0.136
ot2	8.423	3.571	2.308
ot3	10.415	1.480	7.036
Observational Choice PE	-1.551	0.816	-1.900
ot1: Observational Choice PE	-17.131	5.419	-3.161
ot2: Observational Choice PE	-15.253	4.279	-3.564
ot3: Observational Choice PE	-5.874	1.898	-3.095

SI Table. 4. Parameter estimates of the reactive pupil dilation response in the 2 s time-window following the presentation of the demonstrator's choice.

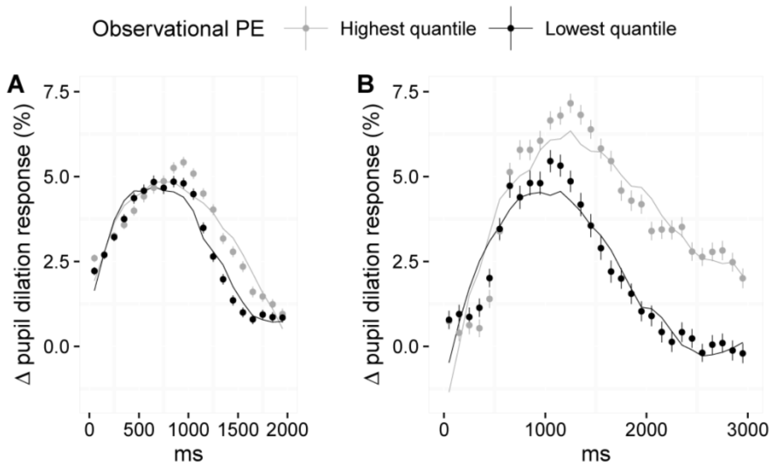

SI Fig. 3. A. Reactive pupil dilation responses are larger when the demonstrator makes a more unexpected choice based on the probabilities that the participant would make either choice at that point in time, calculated as the Observational Choice Prediction Error. B. Reactive pupil dilation responses following the signal indicating that the demonstrator received a shock are greater when shocks are more unexpected, calculated as the absolute Observational Prediction Error. Data points show empirical data and lines show model fits using growth curve analysis. Grey points and lines correspond to the observational PEs within the highest quantile while black points and lines correspond to the observational PEs within the lowest quantile.

The reactive pupil dilation responses when participants heard the signal indicating that the demonstrator received a shock were greater when the (model derived) absolute observational prediction error was large, i.e. more surprising, see SI Fig. 3 B. The effect of the absolute observational prediction error was significant ($\chi^2(1) = 11.24$, $p<0.01$)as well as the effect of the absolute observational prediction on the linear and quadratic time terms(ot1:Absolute Observational PE: $\chi^2(1) = 10.81$, $p<0.01$; ot2: Absolute Observational PE: $\chi^2(1) = 7.42$, $p<0.01$). In addition there were significant effects of the linear, quadratic and cubic time terms (ot1: $\chi^2(1) = 13.30$, $p<0.01$; ot2: $\chi^2(1) = 59.12$, $p<0.01$; ot3: $\chi^2(1) = 81.55$, $p<0.01$). See SI table 5 for parameter estimates.

	Estimates	Std.Error	t
Intercept	1.182	0.602	1.963
ot1	-6.095	1.247	-4.889
ot2	-5.537	1.151	-4.810
ot3	5.256	0.738	7.123
Absolute Observational PE	1.449	0.373	3.884
ot1: Absolute Observational PE	2.268	0.902	3.289
ot2: Absolute Observational PE	-1.763	0.647	-2.724
ot3: Absolute Observational PE	-0.470	0.511	-0.919

SI Table. 5. Parameter estimates of the reactive pupil dilation response in the 2 s time-window following the presentation of the demonstrator's choice.

5.4. Gaze patterns

Previous studies on gaze direction during viewing of emotional pictures have shown that humans initially direct visual attention towards both pleasant and unpleasant/threatening (6), as compared to neutral (7), stimuli. Studies on gaze direction during decision making has shown that gaze direction predicts the preferred choice (8), and more so the closer in time to the decision (9). To the best of our knowledge, no one has studied gaze direction during own or a demonstrator's choice decision in an avoidance task. Analyses of gaze patterns during stimuli presentations using logistic GCA with Areas of Interest (AOIs; optimal choice/suboptimal choice) as response variables showed that participants directed their gaze more towards the optimal compared to the suboptimal choice during both the Demonstrator and Individual Phase, see SI fig. 4A. These results show that gaze can be used as a measure of learning and support previous studies showing that gaze is directed towards the preferred choice (8) as well as extending these findings by showing that this is the case both during own and other´s choice.

Reinforcement learning modeling allowed us to make specific predictions about participants' gaze behavior during observation of available choices by incorporating trial-by-trial beliefs about which stimulus is the optimal one to choose. We did this by incorporating into our

statistical analysis a model-derived measure of the participants' certainty at each trial of which choice would lead to avoidance of shock (calculated as the difference in expected values, see SI 3.3. for details), which improved our models significantly ($p<0.001$, during both the demonstrator and individual phase). As predicted, participants who were more certain that the optimal compared to the suboptimal choice would lead to avoidance of shock were more probable to direct their gaze towards the optimal choice over time compared to those that were less certain, see SI Fig. 4B, further supporting the hypotheses that gaze is directed towards the (believed) optimal choice rather than the dangerous/aversive during both observed and own choices (8).

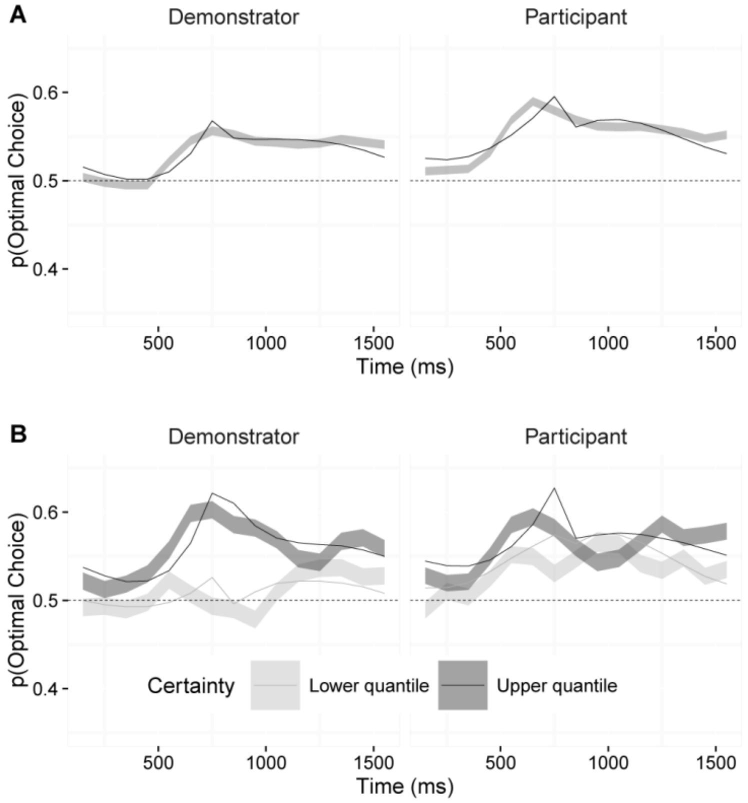

SI Fig.4. A. The probability of participants directing gaze towards the optimal compared to the suboptimal choice following the onset of stimuli presentation. B. The probability of directing gaze towards the optimal choice was modulated by the RL model-derived certainty (difference in expected value between optimal and suboptimal choice) at each trial. Participants directed their gaze more towards the optimal choice when they were more certain that that choice was better than the other. For the trials within the highest quantile of certainty, the probability of directing gaze toward the optimal choice exceeded 0.6. For the trials within the lowest quantile of certainty, the probability of directing gaze toward the optimal choice remained around chance. Grey ribbons show actual data within one standard deviation from the mean while the black lines represent the model derived probabilities. The dotted line indicates the chance probability, 0.5.

5.5. Skin conductance responses

To investigate psychophysiological measures of observational learning and further validate our RL model we analyzed the effect of the observational prediction errors on skin conductance responses, SCRs, following observation of choice and upon hearing that the demonstrator received a shock. SCR is commonly used as a measure of autonomic sympathetic arousal (10) shown to covary with pupil dilation responses (11) and is used as an index of learning in classical conditioning (12) and decision making tasks (13). We expect higher SCRs following more surprising events (i.e. larger absolute prediction errors) since these should be more arousing. As predicted we saw a significant effect of the observational Choice PE on SCRs following the presentation of the demonstrators choices ($\chi^2(1) =5.84, p=0.02$) as well as an effect of the observational PE on SCRs following the signal indicating that the demonstrator received a shock ($\chi^2(1) =15.539, p<0.01$). SCRs were larger following greater observational PEs (observational Choice PE: $\beta=0.04, SE=0.02, t=2.42$; Observational PE: $\beta=0.13, SE=0.03, t=3.94$).

5.6. Parameter analyses

We used the winning model (above, SI 4.2.) to investigate patterns of parameter distribution. To do this we first used cluster analysis using a Gaussian finite mixture model fitted by an estimation-maximization (EM) algorithm (mclust package) to detect three clusters of parameter combinations: i) low values of both α and β, ii) medium to high values of α and medium to low values of β, iii) medium to high values of α and high values of β (see SI Fig 5). Fitted parameters from participants in the Described-High group were categorized as belonging mainly to cluster i and ii (DH; i:10, ii:9, iii:2) while fitted parameters from participants in the Described-Low group were categorized as belonging mainly to cluster ii (DL; i:5, ii:16, iii:1). Although close to trending this difference between groups was not significant (p=0.16). However, if we categorized participants using a median split of mean overall performance there was a significant difference between high performing (mean performance > 0.8)

and low performing (mean performance < 0.8) participants (χ^2(NA)=10.34, p=0.004, simulated p-value) where fitted parameters of high performers mainly were categorized as belonging to cluster i (high performers; i:12, ii; 9, iii:0) while those of low performers were mainly categorized as belonging to cluster ii (low performers; i: 3, ii:16, iii:3).

5.7. Questionnaires

We saw no significant differences in personality traits or level of anxiety between the groups (STAI-state: *t(40.62)=0.44,p=0.67*; STAI-trait: *t(38.86)=-1.00, p=0.33;* Empathic concern: *t(40.74)=0.48,p=0.63;* Fantasy: *t(40.02)=0.99,p=0.33;* Perspective taking: *t(39.90)=0.01,p=0.99;* Conformity: *t(40.73)=0.08,p=0.93*)

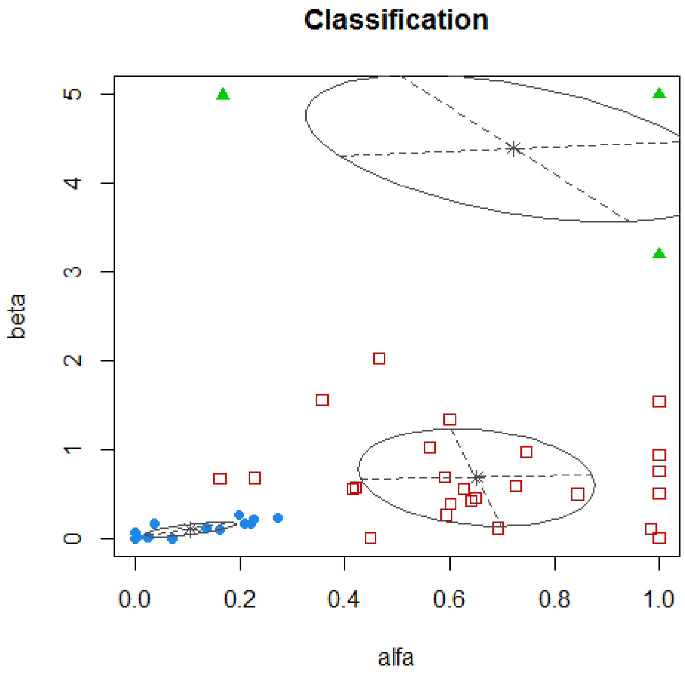

SI Fig. 5. The distribution of fitted parameters in the winning model and how they are classified based on cluster analysis; blue = cluster i, red = cluster ii, green = cluster iii, see SI Results 5.6.

7. SI References

1. Watkins CJCH, Dayan P (1992) Q-Learning. *Mach Learn* 8:279–292.

2. Mirman D (2014) *Growth Curve Analysis and Visualization Using R Analysis and Visualization Using R*.

3. Barr DJ, Levy R, Scheepers C, Tily HJ (2013) Keep it maximal. *J Mem Lang* 68(3):1–43.

4. R Core Team (2016) R: A language and environment for statistical computing. Available at: https://www.r-project.org/.

5. Wagenmakers E-J, Farrell S (2004) AIC model selection using Akaike weights. *Psychon Bull Rev* 11(1):192–6.

6. Calvo MG, Avero P (2005) Time course of attentional bias to emotional scenes in anxiety: Gaze direction and duration. *Cogn Emot* 19(3):433–51.

7. Calvo MG, Lang PJ (2004) Gaze Patterns When Looking at Emotional Pictures : Motivationally Biased Attention 1. 28(3).

8. Shimojo S, Simion C, Shimojo E, Scheier C (2003) Gaze bias both reflects and influences preference. *Nat Neurosci* 6(12):1317–22.

9. Krajbich I, Armel C, Rangel A (2010) Visual fixations and the computation and comparison of value in simple choice. *Nat Neurosci* 13(10):1292–8.

10. Critchley HD, Elliott R, Mathias CJ, Dolan RJ (2000) Neural activity relating to generation and representation of galvanic skin conductance responses: a functional magnetic resonance imaging study. *J Neurosci* 20(8):3033–3040.

11. Bradley MM, Miccoli L, Escrig MA, Lang PJ (2008) The pupil as a measure of emotional arousal and autonomic activation. *Psychophysiology* 45(4):602–607.

12. Öhman A, Mineka S (2001) Fears, phobias, and preparedness: Toward an evolved module of fear and fear learning. *Psychol Rev* 108(3):483–522.

13. Dawson ME, Schell AM, Courtney CG (2011) The skin conductance response, anticipation, and decision-making. *J Neurosci Psychol Econ* 4(2):111–116.
